# Prediction of clinically significant complication following neoadjuvant chemoimmunotherapy in resectable esophageal cancer: a dynamic systemic inflammatory biomarker-based model

**DOI:** 10.3389/fimmu.2026.1719339

**Published:** 2026-03-25

**Authors:** Maohui Chen, Zhenyuan Yang, Yizhou Huang, Hongmu Li, Taidui Zeng, Shuliang Zhang, Guanglei Huang, Chun Chen, Bin Zheng

**Affiliations:** 1 Department of Thoracic Surgery, Fujian Medical University Union Hospital, Fuzhou, China; 2 Key Laboratory of Cardio-Thoracic Surgery (Fujian Medical University), Fujian Province University, Fuzhou, China; 3 National Key Clinical Specialty of Thoracic Surgery, Fuzhou, China; 4 Clinical Research Center for Thoracic Tumors of Fujian Province, Fuzhou, China

**Keywords:** dynamic inflammatory biomarkers, esophageal cancer, neoadjuvant chemoimmunotherapy, postoperative complications, predictive model

## Abstract

**Introduction:**

Neoadjuvant chemoimmunotherapy (NICT) shows promise in locally advanced esophageal squamous cell carcinoma (LA-ESCC), yet may induce complex inflammatory-immune changes that complicate postoperative risk stratification. Current static inflammatory indices lack generalizability across populations and timing.

**Methods:**

This single-center retrospective cohort study analyzed 273 patients. Patients were stratified by Clavien-Dindo grade into a non-significant complication (NSC) group (CD < II) and a clinically significant complication (CSC) group (CD ≥ II). Dynamic (Δ) inflammatory indices were constructed from pre- and post-NICT hematologic markers. Optimal cutoffs were determined via ROC analysis and the Youden index, after which each Δ indicator was transformed into a dichotomous variable. Significant predictors from univariable logistic regression were incorporated into the multivariable logistic regression, which integrated ΔNPR-SII2 with clinical factors to build a final predictive model.

**Results:**

Of the cohort, 212 and 61 patients were classified into the NSC and CSC groups, respectively. Multivariate logistic regression identified age ≥ 70 years (odds ratio [OR] = 2.37, 95% CI: 1.01–5.55), low body mass index (< 18.5; OR = 2.20, 95% CI: 1.08–4.50), an elevated ECOG score (≥ 2; OR = 3.98, 95% CI: 1.13–14.05), and ΔNPR-SII2 (OR = 8.34, 95% CI: 3.92–17.74) as independent predictors of major complications. The model demonstrated good discriminatory power, with an area under the curve (AUC) of 0.784 (95% CI: 0.724–0.844) and a bootstrap-validated C-index of 0.772. Calibration performance was satisfactory (Brier score = 0.138).

**Discussion:**

We developed and internally validated a dynamic inflammatory biomarker-based model for preoperative prediction of major complications post-NICT. The ΔNPR-SII2 index captures treatment-induced immune-inflammatory dysregulation and provides a clinically translatable tool for risk-adapted perioperative management.

## 1 Introduction

According to the International Agency for Research on Cancer (IARC), esophageal cancer ranks 11th in global incidence and 7th in cancer-related mortality, thereby persisting as a substantial public health burden (1). For patients with locally advanced esophageal squamous cell carcinoma (ESCC), multidisciplinary comprehensive therapy is regarded as the cornerstone of management; notably, neoadjuvant therapy followed by radical resection has been widely adopted and is increasingly standardized (2). Nevertheless, despite iterative advances in perioperative care, esophagectomy remains associated with a high rate of complications that can impede short-term convalescence and, conversely, compromise long-term survival (3, 4). Moreover, neoadjuvant regimens—particularly cytotoxic chemotherapy and immune checkpoint blockade—elicit profound histopathologic and immunologic alterations, thereby introducing additional complexity for perioperative risk stratification and prediction of postoperative outcomes (5).

The systemic inflammatory response is recognized as a pivotal determinant of tumorigenesis, disease progression, and postoperative recovery (6, 7). Numerous investigations have attempted to predict complications and survival after esophagectomy using inflammation-related hematologic indices; however, these evaluations have been largely static, relying on single time-point measurements. Representative ratio-based markers—such as the neutrophil-to-lymphocyte ratio (NLR), lymphocyte-to-monocyte ratio (LMR), and systemic immune-inflammation index (SII)—leverage leukocyte differentials and platelet counts to reflect proinflammatory and immunosuppressive milieus as well as tumor-associated systemic responses (8–11). In parallel, composite inflammation–nutrition scores—including C-reactive Protein to Albumin Ratio (CAR), the Glasgow Prognostic Score (GPS/mGPS), the fibrinogen–albumin (FA) score, and the Prognostic Nutritional Index (PNI)—seek to provide an integrated appraisal of host status from dual inflammatory and nutritional perspectives (12–15). Notably, although these metrics demonstrate prognostic utility across cohorts, their performance is inconsistent across populations, perioperative phases, and treatment settings, and most lack a framework that synthesizes signals from multiple biological pathways.

Neoadjuvant immunotherapy has emerged as a focus of intensive investigation in locally advanced ESCC and has demonstrated promising efficacy. Accumulating evidence indicates that immune checkpoint inhibitors can enhance pathologic regression, promote tumor downstaging, and consequently increase surgical eligibility and optimize perioperative outcomes (16–18). By potentiating antitumor immunity and facilitating malignant-cell recognition and clearance, immunotherapy may also remodel the dynamics of systemic inflammatory and immune-related biomarkers. Therefore, whether and how such evolving markers predict outcomes specifically within neoadjuvant chemotherapy and immunotherapy paradigms remains to be elucidated.

Building on this rationale, the present study was designed to serially quantify inflammatory status during neoadjuvant chemotherapy from a dynamic perspective and to develop a multifactorial predictive model for perioperative outcomes. Key preoperative clinical variables will be integrated with dynamic inflammatory metrics to construct and validate an interpretable nomogram for individualized risk stratification.

## 2 Materials and methods

### 2.1 Patients and study design

This single-center retrospective cohort included consecutive patients with locally advanced esophageal squamous cell carcinoma (LA-ESCC) who underwent neoadjuvant immunochemotherapy (NICT) followed by radical esophagectomy in the Department of Thoracic Surgery, Fujian Medical University Union Hospital, between August 2023 and January 2025. Eligibility criteria were defined *a priori* as (1): pathologically or clinically diagnosed locally advanced esophageal cancer treated with 2–3 cycles of NICT (paclitaxel or nab-paclitaxel plus a PD-1 inhibitor) (2); subsequent curative-intent esophagectomy; and (3) availability of complete perioperative clinical and laboratory data. Exclusion criteria comprised (1): incomplete or missing data essential for analysis and (2) a history of other active malignancies or significant systemic comorbidities expected to independently influence postoperative outcomes. The study was conducted in accordance with the Declaration of Helsinki and received approval from the Ethics Committee of Fujian Medical University Union Hospital.

### 2.2 Data collection and variable definitions

Clinical information was abstracted retrospectively from the electronic medical record using standardized case-report forms. Baseline variables included demographics (age, sex), body mass index (BMI), tobacco and alcohol history, Eastern Cooperative Oncology Group (ECOG) performance status, tumor clinical characteristics, immune phenotyping, pulmonary function variables and baseline inflammatory indices. Perioperative variables comprised surgical approach, operative duration, lymphadenectomy strategy, and postoperative complications. Inflammatory markers were obtained at 2 prespecified time points—prior to NICT initiation and preoperatively (within 7 days before surgery)—from routine hematology and biochemical panels. Indices were calculated as follows: neutrophil-to-lymphocyte ratio (NLR) = neutrophils/lymphocytes; lymphocyte-to-monocyte ratio (LMR) = lymphocytes/monocytes; platelet-to-lymphocyte ratio (PLR) = platelets/lymphocytes; neutrophil-to-platelet ratio (NPR) = neutrophils/platelets; platelet-to-albumin ratio (PAR) = platelets/albumin; systemic immune-inflammation index (SII) = (platelets × neutrophils)/lymphocytes; and systemic inflammation response index (SIRI) = (neutrophils × monocytes)/lymphocytes. Dynamic change (Δ) for each marker was defined as the relative change from baseline to preoperative measurement: Δ = (Post − Pre)/Pre, where “Pre” denotes the pre-NICT value and “Post” the preoperative value.

### 2.3 Outcome measures

The primary outcome was the severity of postoperative complications within 30 days of surgery or through index hospitalization, whichever was longer. Complications were graded using the Clavien–Dindo (CD) classification; for patients with multiple events, the highest grade was used for analysis. Patients were stratified into 2 categories: the non-significant complication (NSC) group (CD grade < II; none or grade I) and the clinically significant complication (CSC) group (CD grade ≥ II; events requiring pharmacologic or more invasive interventions). This dichotomization was selected *a priori* to reflect clinically meaningful therapeutic escalation.

### 2.4 Statistical analysis

All analyses were performed using R, version 4.3.1 (R Foundation for Statistical Computing). Two-sided P values < 0.05 were considered statistically significant, and 95% CIs were reported where applicable. Continuous variables with approximately normal distributions were summarized as mean ± SD and compared using the independent-samples t test; non-normally distributed variables were summarized as median (IQR) and compared using the Mann–Whitney U test. Categorical variables were presented as No. (%) and compared using the χ² test or Fisher exact test, as appropriate. Diagnostic performance for each Δ index was evaluated using receiver operating characteristic (ROC) analysis, and optimal cutoffs were identified by maximizing the Youden index (sensitivity + specificity − 1). Each Δ marker was then dichotomized at its optimal threshold to generate a binary variable (hereafter “Δ-binary”: 0 for values ≤ cutoff; 1 for values > cutoff). The univariate analysis in this study incorporated the following patient and clinical characteristics: age (≥70 years), low BMI (<18.5), ΔNPR-SII2, ECOG (≥2), sex, extent of lymphadenectomy fields, surgical approach, smoking history, drinking history, hypertension, diabetes, tumor location, clinical T stage (cT), clinical N stage (cN), and operation duration. Univariable logistic regression was first used to screen candidate predictors of CSC status, after which variables with P < 0.05 were entered into a multivariable logistic regression model to estimate adjusted odds ratios (ORs) with 95% CIs and to identify independent factors associated with complication-grade escalation. The continuous composite indicator was constructed by assigning stepwise regression-derived weights to significant binary predictors. This was followed by dichotomization to yield the final binary indicator. Subsequently, a predictive model integrating this composite index with preoperative covariates was constructed via logistic regression, with discrimination quantified by the area under the ROC curve (AUROC) and corresponding 95% CIs, and calibration assessed by bootstrap-corrected calibration plots. A nomogram was generated to facilitate bedside application and individualized risk estimation. Internal validation was performed to evaluate robustness and mitigate optimism bias. Therefore, the final tool is interpretable and clinically actionable for perioperative risk stratification in LA-ESCC.

## 3 Results

### 3.1 Baseline patient characteristics

This study ultimately enrolled a total of 273 eligible patients with locally advanced esophageal cancer. All patients received neoadjuvant chemoimmunotherapy followed by radical esophagectomy. Based on the Clavien-Dindo classification for postoperative complications, the cohort was stratified into the NSC group (Clavien-Dindo grade < II; n=212) and the CSC group (Clavien-Dindo grade ≥ II; n=61). The baseline characteristics of the two patient groups are presented in **Table 1**. Comparative analyses revealed significant between-group differences in specific preoperative inflammatory markers (post-NLR, post-NPR, post-SII, post-SIRI) and ECOG status (stratified as ≥2 *vs*. ≤1). All other baseline and perioperative variables remained comparable between the two groups. Postoperative complications differed significantly between the two groups (**Table 2**). The most frequent complications were pneumonia and anastomotic leakage, which constituted 29.30% (80/273) and 10.99% (30/273) of all cases, respectively. Notably, a majority of these specific complications were graded as clinically significant: 56.25% (45/80) of pneumonias and 70.00% (21/30) of anastomotic leaks reached a severity of CD ≥ II. Specifically in the CSC cohort, pneumonia was the predominant complication (73.77%), followed by anastomotic leak (34.43%) and arrhythmia (14.75%). An overview of the major immune-related adverse events observed in this cohort, including event categories and corresponding severity grades, is provided in **Supplementary Table 1**.

**Table 1:** Baseline characteristics of the two groups of patients.

Characteristics	NSC group (n=212)	CSC group (n=61)	p-value
Age, Median (Q1, Q3)	61.00 (57.00, 67.00)	61.00 (58.00, 69.00)	0.303
Sex, n (%)			0.948
Male	173 (81.60)	50 (81.97)	
Female	39 (18.40)	11 (18.03)	
BMI, Median (Q1, Q3)	21.45 (19.49, 23.28)	20.98 (18.43, 22.58)	0.413
Smoking, n (%)			0.585
Never	74 (34.91)	19 (31.15)	
Current or former	138 (65.09)	42 (68.85)	
Drinking, n (%)			0.210
Never	92 (43.40)	21 (34.43)	
Current or former	120 (56.60)	40 (65.57)	
Hypertension, n (%)			0.560
Never	164 (77.36)	45 (73.77)	
Current or former	48 (22.64)	16 (26.23)	
Diabetes, n (%)			0.387
Never	200 (94.34)	55 (90.16)	
Current or former	12 (5.66)	6 (9.84)	
FEV1%pred			0.803
≥90%	173 (81.60)	48 (78.69)	
70–90%	32 (15.09)	10 (16.39)	
<70%	7 (3.30)	3 (4.92)	
ECOG, n (%)			0.015
≤1	204 (96.23)	53 (86.89)	
≥2	8 (3.77)	8 (13.11)	
Tumor Location, n (%)			0.833
Upper thoracic	19 (8.96)	7 (11.48)	
Middle thoracic	116 (54.72)	33 (54.10)	
Lower thoracic	77 (36.32)	21(34.43)	
cT, n (%)			0.950
2	52 (24.53)	15 (24.59)	
3	132 (62.26)	37 (60.66)	
4	28 (13.21)	9 (14.75)	
cN, n (%)			0.413
0	29 (13.68)	12 (19.67)	
1	85 (40.09)	18 (29.51)	
2	73 (34.43)	24 (39.34)	
3	25 (11.79)	7 (11.48)	
PD-L1 expression			0.815
CPS ≥10	55 (25.94)	18 (29.51)	
CPS < 10	98 (46.23)	28 (45.90)	
Unknown	59 (27.83)	15 (24.59)	
Pre-NLR, Median (Q1, Q3)	2.46 (1.83.3.46)	2.57 (2.13,3.05)	0.654
Pre-LMR, Median (Q1, Q3)	4.07 (3.09.5.59)	3.83 (3.27. 5.00)	0.440
Pre-PLR, Median (Q1, Q3)	154.76(120.59,187.21)	152.13 (107.69,192.21)	0.611
Pre-NPR, Median (Q1, Q3)	0.02 (0.01.0.02)	0.02 (0.01.0.02)	0.187
Pre-PAR, Median (Q1, Q3)	6.04 (4.93. 7.05)	5.95 (4.69.7.81)	0.886
Pre-SII, Median (Q1, Q3)	655.74(415.70,882.96)	644.21(438.04,885.85)	0.768
Pre-SIRI, Median (Q1, Q3)	1.00 (0.62. 1.56)	1.01 (0.81. 1.49)	0.313
Post-NLR, Median (Q1, Q3)	2.22 (1.66, 3.03)	3.40 (2.53. 4.57)	<0.001
Post-LMR, Median (Q1, Q3)	3.83 (2.86.4.74)	3.63 (2.92.5.15)	0.922
Post-PLR, Median (Q1, Q3)	136.38 (104.07,178.10)	129.44(100.60,177.08)	0.846
Post-NPR, Median (Q1, Q3)	0.02 (0.01.0.02)	0.02(0.02.0.03)	<0.001
Post-PAR, Median (Q1, Q3)	5.05 (4.16.6.27)	5.19 (4.29,6.02)	0.957
Post-SII, Median (Q1, Q3)	470.50(329.12,663.76)	709.05 (477.81.986.35)	<0.001
Post-SIRI, Median (Q1, Q3)	0.92 (0.64, 1.42)	1.41 (0.94, 1.75)	<0.001

Q_1_, 1st Quartile; Q_3_, 3rd Quartile; BMI, Body Mass Index; ECOG, Eastern Cooperative Oncology Group.

**Table 2:** Perioperative data and postoperative complications in the two groups of patients.

Characteristics	NSC group (n=212)	CSC group (n=61)	p-value
Operative duration (min)	339.00 (304.75, 374.00)	331.00 (310.00, 382.00)	0.601
Lymphadenectomy field(s), n (%)			0.780
2	187 (88.21)	53 (86.89)	
3	25 (11.79)	8 (13.11)	
Surgical procedure, n (%)			0.220
VATS	159 (75.00)	42 (68.85)	
RATS	50 (23.58)	16 (26.23)	
Open	3 (1.42)	3 (4.92)	
Anastomotic fistula, n (%)			<0.001
No	203 (95.75)	40 (65.57)	
Yes	9 (4.25)	21 (34.43)	
Pneumonia, n (%)			<0.001
No	177 (83.49)	16 (26.23)	
Yes	35 (16.51)	45 (73.77)	
Arrhythmia			0.114
No	195 (91.98)	52 (85.25)	
Yes	17 (8.02)	9 (14.75)	
Chylothorax			0.011
No	212 (100.00)	58 (95.08)	
Yes	0 (0.00)	3 (4.92)	
ICU, n (%)			<0.001
No	212 (100.00)	56 (91.80)	
Yes	0 (0.00)	5 (8.20)	
Secondary operation, n (%)			0.073
No	212 (100.00)	59 (96.72)	
Yes	0 (0.00)	2 (3.28)	

### 3.2 Diagnostic efficacy of dynamic inflammatory indicators and identification of new indicators

**Figure 1:**
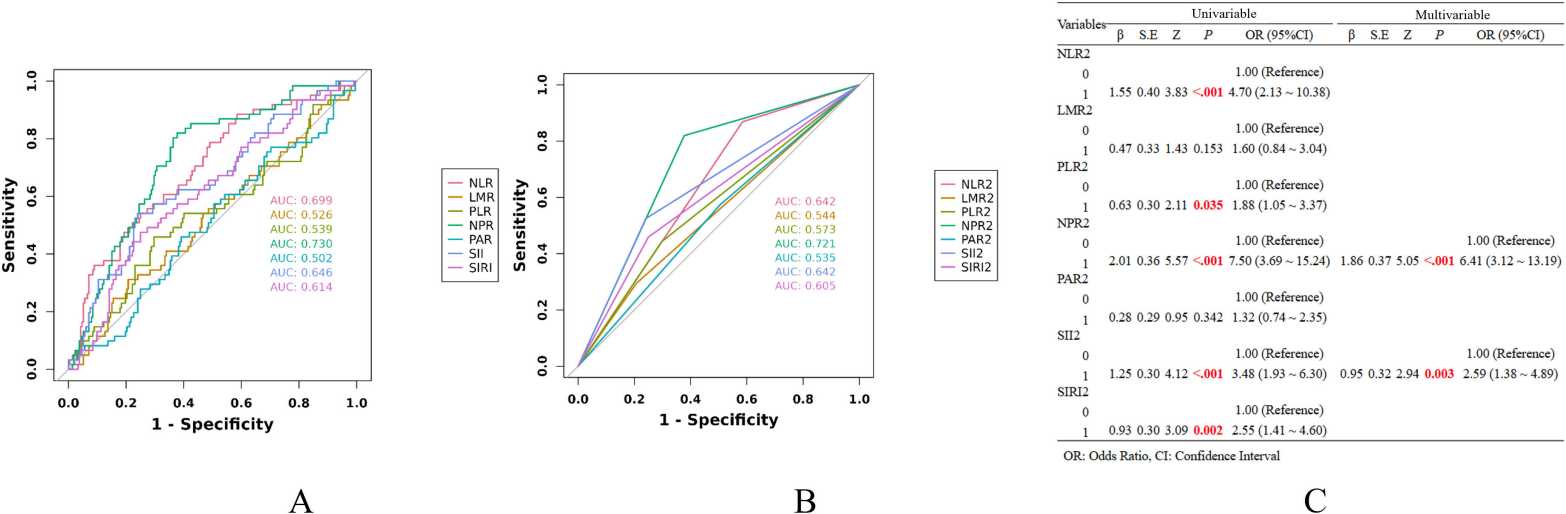
**(A)** ROC curve analysis for each Δ metric; **(B)** ROC curve analysis for each Δ2 metric; **(C)** Univariable and multivariable logistic regression analysis for Δ2 metrics.

To evaluate the predictive performance of the Δ metrics for postoperative complication grade escalation, we initially conducted receiver operating characteristic (ROC) curve analyses for each inflammatory indicator (**Figure 1A**). As summarized in **Table 3**, the optimal cutoff value for each metric was determined by maximizing the Youden index. Subsequently, each Δ indicator was dichotomized based on its respective cutoff: values below the threshold were assigned a score of 0, while those equal to or above it were assigned a score of 1, thereby generating a new binary variable, designated as the Δ2 indicator. The predictive performance of each Δ2 indicator was assessed using ROC curve analysis (**Figure 1B**). Variables with a P-value < 0.05 in the univariable logistic regression were subsequently included in a multivariable logistic model. A bidirectional stepwise regression approach was applied to identify independent predictors, leading to the development of a novel composite indicator termed ΔNPR-SII. This index was calculated as 1.86 × ΔNPR2 + 0.95 × ΔSII2 (**Figure 1C**). The continuous variable ΔNPR-SII was transformed into a binary indicator (ΔNPR-SII2) according to the rule: a score of 0 for values below the optimal cutoff and a score of 1 for values at or above the optimal cutoff.

**Table 3:** Analysis of ROC curves for each indicator.

Index	AUC(95% CI)	Cut off	Sensitivity	Specificity	Youden index	
ΔNLR	0.699 (0.624-0.773)	-0.177	0.885	0.415	0.300
ΔLMR	0.526 (0.442-0.610)	0.260	0.311	0.792	0.103
ΔPLR	0.539 (0.453-0.626)	0.085	0.443	0.703	0.146
ΔNPR	0.730 (0.662-0.798)	0.100	0.820	0.623	0.443
ΔPAR	0.502 (0.419-0.585)	-0.164	0.574	0.495	0.069
ΔSII	0.646 (0.567-0.726)	0.119	0.541	0.759	0.300
ΔSIRI	0.614 (0.534-0.694)	0.493	0.475	0.750	0.225
ΔNLR2	0.642 (0.588-0.696)		0.869	0.415	0.284
ΔLMR2	0.544 (0.480-0.608)		0.295	0.792	0.087
ΔPLR2	0.573 (0.503-0.643)		0.443	0.703	0.146
ΔNPR2	0.721 (0.663-0.780)		0.820	0.623	0.443
ΔPAR2	0.535 (0.463-0.606)		0.574	0.495	0.069
ΔSII2	0.642 (0.573-0.711)		0.525	0.759	0.284
ΔSIRI2	0.605 (0.535-0.674)		0.459	0.750	0.209
ΔNPR-SII	0.768 (0.706-0.829)	1.405	0.820	0.623	0.443
ΔNPR-SII2	0.721 (0.663-0.780)		0.820	0.623	0.443

### 3.3 Predictive modelling and effectiveness assessment

**Figure 2:**
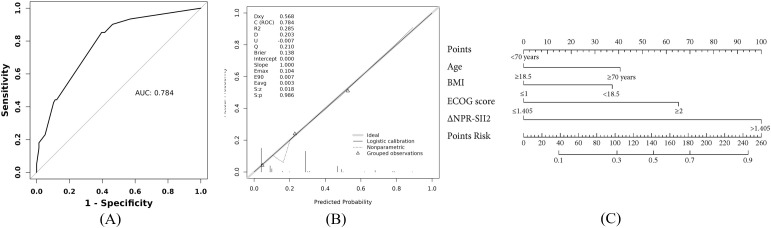
**(A)** ROC curve; **(B)** Calibration curve; **(C)** Column line graph.

A predictive model was developed through univariable and multivariable logistic regression analyses involving ΔNPR-SII2 and additional preoperative factors. In univariate analyses, age (≥70 years), low body mass index (<18.5), high ECOG score (≥2), and ΔNPR-SII2 emerged as potential predictors (p < 0.05). These significant variables were subsequently incorporated into a multivariate logistic regression model for further evaluation (**Table 4**). The multivariate analysis confirmed that the same factors independently predicted more severe complications. Of particular significance, patients with a ΔNPR-SII2 score of 1 had an 8.34-times greater risk of experiencing a high-grade complication (OR = 8.34, 95% CI: 3.92–17.74, p < 0.001) than those with a score of 0, thereby identifying a high-risk patient subgroup. The final model was evaluated through its ROC curve, calibration plot, nomogram, and internal bootstrap validation, as shown in **Figure 2**. The ROC analysis yielded an AUC of 0.784 (95% CI: 0.724–0.844). Assessment of the calibration curve provided the following performance measures: Brier score = 0.138, Intercept = 0.000, Slope = 1.000, Emax = 0.104, E90 = 0.007, Eavg = 0.003. The model’s discriminative power, indicated by a C-statistic of 0.784, was consistent with the AUC. Internal validation was conducted using the bootstrapping method with 1,000 resamples to evaluate the model’s robustness. The validation results demonstrated a Harrell’s C-index of 0.772 and a standard deviation of the ROC of 0.043.

**Table 4:** Univariate and multivariate analysis of influencing factors (logistic regression).

Variables	Univariable	Multivariable		
	p-value	OR	95% CI	p-value
Age (≥70 years)				
No	reference			
Yes	0.008	2.37	1.01 – 5.55	0.048
Low BMI (<18.5)				
No	reference			
Yes	0.006	2.20	1.08 – 4.50	0.030
ΔNPR-SII2				
≤1.405	reference			
>1.405	<0.001	8.34	3.92 – 17.74	<0.001
ECOG				
≤1	reference			
≥2	0.010	3.98	1.13 – 14.05	0.032
Sex				
Female	reference			
Male	0.948			
Lymphadenectomy field(s), n (%)				
2 fields	reference			
3 fields	0.780			
Surgical procedure				
VATS	reference			
RATS	0.568			
Open	0.111			
Smoking				
Never	reference			
Current or former	0.585			
Drinking				
Never	reference			
Current or former	0.211			
Hypertension				
Never	reference			
Current or former	0.560			
Diabetes				
Never	reference			
Current or former	0.253			
Tumor Location				
Upper thoracic	reference			
Middle thoracic	0.593			
Lower thoracic	0.552			
cT				
2	reference			
3	0.934			
4	0.823			
cN				
0	reference			
1	0.119			
2	0.580			
3	0.476			
Operation duration	0.633			

CI, Confidence Interval; OR, Odds Ratio.

## 4 Discussion

This study developed and internally validated a multivariable logistic regression model to preoperatively estimate the risk of clinically significant postoperative complications (Clavien-Dindo grade ≥ II) in resectable esophageal cancer after NICT. Dynamic systemic inflammatory biomarkers were integrated with clinical covariates to enable individualized risk stratification. Notably, four variables—age ≥ 70 years, low BMI, ECOG performance status, and the composite dynamic index ΔNPR-SII2—were incorporated as key predictors. The model demonstrated favorable discrimination and calibration on internal validation, and its translation into a nomogram renders it readily applicable at the bedside for risk communication and shared decision-making.

The principal methodological strength lies in the composite and dynamic design. Logistic regression was used to identify independent predictors while accounting for potential confounding. For each dynamic (Δ) marker, thresholds were determined objectively via receiver operating characteristic (ROC) analysis using the Youden index; subsequent dichotomization enhanced clinical interpretability. A staged analytical strategy was adopted: univariable screening of Δ-based binary indicators (two-sided α = 0.05) was followed by multivariable modeling to elucidate adjusted associations. This process yielded the original dynamic inflammation metric, ΔNPR-SII2, which integrates neutrophil-to-platelet ratio (NPR) and systemic immune-inflammation index (SII) to capture treatment-related shifts in pro-inflammatory activity and immune tone. Therefore, the approach addresses limitations of static measurements by emphasizing longitudinal change.

In this study, inflammatory markers were measured at two time points: before the initiation of neoadjuvant therapy and preoperatively (within 7 days). The longitudinal change (Δ) captures the systemic evolution of inflammation and immune status from baseline to the immediate preoperative period. This trajectory likely results from the combined effects of multiple factors, including chemotherapy-induced bone marrow suppression and tissue injury-related inflammation, tumor-driven systemic inflammatory responses, as well as the patient’s physiological reserve and stress adaptation. Therefore, ΔNPR-SII2 is considered a composite marker reflecting “treatment-associated systemic inflammatory/immune dysregulation”. Its clinical utility lies in integrating and quantifying the extent of physiological perturbation experienced by patients undergoing multimodal therapy, thereby helping to identify those at increased perioperative risk. The primary aim of this study is to validate its predictive performance as a practical risk-stratification tool, rather than to elucidate immunotherapy-specific mechanistic pathways.

Biological plausibility supports the weighting of ΔNPR-SII2. Neoadjuvant immunotherapy can potentiate effector T-cell responses while concurrently provoking systemic inflammation and myeloid-driven immunosuppression, reflecting a bidirectional immune milieu (19, 20). The convergent activation of neutrophils and platelets may amplify inflammatory mediator release, endothelial perturbation, and procoagulant pathways, thereby impairing tissue repair and increasing infectious susceptibility (20–23). When coupled with relative lymphocyte depletion, immune homeostasis may be further disrupted (24–26). An elevated ΔNPR-SII2 thus plausibly reflects a dysregulated, pro-inflammatory phenotype with treatment-related immune exhaustion, a context that is mechanistically compatible with pulmonary infections and anastomotic complications. Consequently, early recognition of this phenotype can inform tailored perioperative measures—such as optimization of anti-infective prophylaxis, respiratory rehabilitation, nutritional support, and intensified monitoring. In the final multivariable model, ΔNPR-SII2 emerged as an independent predictor with a larger effect size than the other three covariates (odds ratio [OR] = 8.34 *vs* ORs = 2.20, 2.37, and 3.98, respectively), underscoring its relative contribution to risk estimation. The model containing only ΔNPR-SII2 achieved an AUC of 0.721 (95% CI: 0.663–0.780). In contrast, the comprehensive multivariate model, which incorporated age ≥70 years, low BMI (<18.5), ECOG ≥2, and ΔNPR-SII2, improved the AUC to 0.784 (95% CI: 0.724–0.844). These findings suggest that while ΔNPR-SII2 alone is a robust predictor, integrating key clinical characteristics—such as baseline physiological reserve, nutritional status, and functional capacity—yields a more comprehensive and discriminative risk assessment. Consistent with this, the nomogram assigns the broadest scoring range to ΔNPR-SII2, indicating a predominant influence on total points. Within a total scale of 260 points, an abnormal ΔNPR-SII2 (score = 1) contributes 100 points, highlighting its weight in the calculation. The “Total Points-to-Risk” mapping indicates that even patients with favorable age, nutritional status, and functional scores may retain a nontrivial baseline probability (approximately 30%) of major complications when adverse dynamic inflammatory shifts are present. Although the high OR for ΔNPR-SII2 (OR 8.34, 95% CI 3.92–17.74) indicates a dominant contribution in this cohort. It should be noted that this finding may be partly attributable to residual confounding from unmeasured variables (e.g., subclinical preoperative infections or treatment heterogeneity) or to shared variance with the broader construct of physiological frailty, which is also captured by age, BMI, and ECOG status. These observations should be interpreted as hypothesis-generating rather than definitive, pending external validation. ROC analysis yielded an AUC of 0.784 for the original model, consistent with good discrimination. Internal validation using 1,000 bootstrap resamples produced a C-index of 0.772 with limited variability (SD = 0.043), suggesting stability and reduced optimism. Calibration curves showed close agreement between observed and predicted risks, with points distributed near the 45° line.

For patients identified as high-risk based on model scoring, an enhanced preoperative assessment and optimization protocol is advised. Recommended measures include, but are not limited to, an intensive preoperative pulmonary rehabilitation program, early nutritional assessment and support with particular attention to individuals with low BMI, more thorough screening for occult infections or treatment-related toxicities, and implementation of proactive postoperative monitoring and pulmonary complication prevention strategies such as early ambulation and strict adherence to incentive spirometry. Importantly, it must be emphasized that, pending prospective validation, this model should not be used as the sole basis for altering or discontinuing established neoadjuvant therapy protocols. Rather, it should serve as a complementary tool within multidisciplinary team discussions and be integrated into a comprehensive clinical evaluation framework.

Although the continuous ΔNPR-SII indicator demonstrated marginally higher discriminatory ability (AUC = 0.768 *vs*. 0.721), we ultimately selected its binary form (ΔNPR-SII2) for inclusion in the final clinical prediction model. This decision was based on two principal considerations: first, clinical practicality—binary stratification into high- *vs*. low-risk categories is more intuitive and readily applicable in bedside decision-making, enabling rapid identification of patients who may benefit from intensified intervention, which aligns with the study’s objective of developing a pragmatic tool. Second, model stability—given the limited sample size, incorporating a continuous variable could increase model complexity and potentially compromise the robustness of parameter estimates. We acknowledge that dichotomization results in some loss of information. Future studies with larger cohorts may further evaluate models using continuous or ordinal representations of ΔNPR-SII, or employ more advanced machine-learning techniques to preserve its full informational value—an important direction for subsequent research.

The proposed framework also addresses two recurrent challenges in the literature. First, reliance on single-time-point inflammatory indices (e.g., NLR, PLR, SII, SIRI, CAR, and PNI) has yielded heterogeneous results across cohorts, perioperative windows, and cutoffs, limiting generalizability. By integrating multiple markers into the composite ΔNPR-SII2 metric, the statistical burden of multiple comparisons is reduced and a standardized, prospectively testable measure is provided. Second, conversion of the regression into a nomogram facilitates immediate clinical use and transparent communication of individualized risk on a continuous scale (27). Therefore, the tool is positioned as a pragmatic adjunct rather than a replacement for comprehensive clinical evaluation.

Several limitations warrant emphasis. The single-center, retrospective design introduces risks of selection bias and residual confounding. Thresholds for Δ indicators and the ΔNPR-SII2 coefficient were derived from the present dataset; external, multicenter prospective validation is required to evaluate transportability. We emphasize that, while internal validation was performed using bootstrapping, this does not substitute for external validation in an independent, multicenter, prospective cohort. Prior to clinical implementation, our model must undergo rigorous external validation to confirm its generalizability and to mitigate the risks of overfitting inherent in single-center, retrospective study designs. Moreover, multi-omics features (e.g., radiogenomics, circulating cytokine profiles, or microbiome signatures) were not incorporated; future work could elucidate whether multimodal integration improves sensitivity and generalizability. Finally, although clinically significant complications (Clavien-Dindo ≥ II) were used as the primary endpoint, additional outcomes—such as severe complications (≥ III), readmission, or long-term survival—should be assessed to determine robustness across endpoints and to clarify clinical utility within broader decision pathways. It should be noted that the CSC cohort primarily consists of pneumonia cases. This indicates that the present model may be more relevant for predicting perioperative risks related to esophagectomy—particularly pulmonary complications—rather than outcomes driven by specific mechanisms of immunotherapy. The baseline pulmonary function and other pulmonary risk variables are not included and should be considered for future model refinement and for complication-specific modeling. Furthermore, the dataset lacks immunotherapy-specific pharmacodynamic or mechanistic measurements (e.g., tumor immune infiltration, cytokine profiling, T-cell receptor repertoire dynamics, treatment-response stratification or histopathology of immune-related adverse events). Consequently, it cannot be concluded that ΔNPR-SII2 serves as a specific biomarker for PD-1 inhibitor activity. This study did not conduct stratified analyses to examine the effects of different neoadjuvant treatment regimens—such as paclitaxel versus nab-paclitaxel, or among various PD-1 inhibitors. Although the regimens were largely uniform according to the inclusion criteria, potential heterogeneity among them may have influenced the observed dynamic changes in inflammatory markers as an unmeasured confounder. Owing to the limited sample size and number of events, statistical power was inadequate for meaningful subgroup comparisons. Future studies should prospectively collect detailed treatment data to better assess and adjust for this potential source of confounding.

## 5 Conclusion

This study developed and internally validated a multivariable model that integrates established clinical factors—age, BMI, and ECOG performance status—with a novel dynamic inflammatory index (ΔNPR-SII2) to preoperatively stratify the risk of clinically significant postoperative complications (Clavien-Dindo ≥ II) in esophageal cancer patients receiving NICT. Notably, the model was translated into a nomogram to facilitate bedside application and individualized risk communication, thereby supporting targeted perioperative interventions. Therefore, the tool is positioned as a pragmatic adjunct to clinical judgment and standard pathways rather than a replacement. Prospective, multicenter studies are warranted to externally validate performance, assess transportability, and elucidate the model’s broader clinical utility across additional endpoints.

## Data Availability

The raw data supporting the conclusions of this article will be made available by the authors, without undue reservation.

## References

[1] Bray F Laversanne M Sung H Ferlay J Siegel RL Soerjomataram I . Global cancer statistics 2022: GLOBOCAN estimates of incidence and mortality worldwide for 36 cancers in 185 countries. CA Cancer J Clin. (2024) 74:229–63. doi: 10.3322/caac.21834, PMID: 38572751

[2] Deboever N Jones CM Yamashita K Ajani JA Hofstetter WL . Advances in diagnosis and management of cancer of the esophagus. Bmj. (2024) 385:e074962. doi: 10.1136/bmj-2023-074962, PMID: 38830686

[3] Booka E Takeuchi H Nishi T Matsuda S Kaburagi T Fukuda K . The Impact of Postoperative Complications on Survivals After Esophagectomy for Esophageal Cancer. Med (Baltimore). (2015) 94:e1369. doi: 10.1097/MD.0000000000001369, PMID: 26287423 PMC4616453

[4] Bundred JR Hollis AC Evans R Hodson J Whiting JL Griffiths EA . Impact of postoperative complications on survival after oesophagectomy for oesophageal cancer. BJS Open. (2020) 4:405–15. doi: 10.1002/bjs5.50264, PMID: 32064788 PMC7260404

[5] Postow MA Sidlow R Hellmann MD . Immune-Related Adverse Events Associated with Immune Checkpoint Blockade. N Engl J Med. (2018) 378:158–68. doi: 10.1056/NEJMra1703481, PMID: 29320654

[6] Hanahan D Weinberg RA . Hallmarks of cancer: the next generation. Cell. (2011) 144:646–74. doi: 10.1016/j.cell.2011.02.013, PMID: 21376230

[7] Diakos CI Charles KA McMillan DC Clarke SJ . Cancer-related inflammation and treatment effectiveness. Lancet Oncol. (2014) 15:e493–503. doi: 10.1016/S1470-2045(14)70263-3, PMID: 25281468

[8] Sharaiha RZ Halazun KJ Mirza F Port JL Lee PC Neugut AI . Elevated preoperative neutrophil:lymphocyte ratio as a predictor of postoperative disease recurrence in esophageal cancer. Ann Surg Oncol. (2011) 18:3362–9. doi: 10.1245/s10434-011-1754-8, PMID: 21547702 PMC3192937

[9] Han LH Jia YB Song QX Wang JB Wang NN Cheng YF . Prognostic significance of preoperative lymphocyte-monocyte ratio in patients with resectable esophageal squamous cell carcinoma. Asian Pac J Cancer Prev. (2015) 16:2245–50. doi: 10.7314/APJCP.2015.16.6.2245, PMID: 25824745

[10] Fu X Li T Dai Y Li J . Preoperative systemic inflammation score (SIS) is superior to neutrophil to lymphocyte ratio (NLR) as a predicting indicator in patients with esophageal squamous cell carcinoma. BMC Cancer. (2019) 19:721. doi: 10.1186/s12885-019-5940-6, PMID: 31331297 PMC6647281

[11] Aoyama T Maezawa Y Hashimoto I Esashi R Yamamoto S Kazama K . The Systemic Immune-inflammation Index (SII) Is an Independent Prognostic Factor for Patients With Recurrent Esophageal Cancer After Esophagectomy. In Vivo. (2025) 39:2340–8. doi: 10.21873/invivo.14031, PMID: 40578991 PMC12223646

[12] Aoyama T Maezawa Y Hashimoto I Kazama K Uchiyama M Hara K . The C-reactive Protein to Albumin Ratio (CAR) Is an Independent Prognostic Factor for Recurrence in Patients With Esophageal Cancer After Esophagectomy. Anticancer Res. (2025) 45:3365–72. doi: 10.21873/anticanres.17697, PMID: 40750412

[13] Fujiwara Y Endo S Higashida M Kubota H Yoshimatsu K Ueno T . The prognostic significance of preoperative nutritional/inflammatory markers and clinicopathological features in resectable esophagectomy patients: possibility of nutritional intervention. Esophagus. (2023) 20:234–45. doi: 10.1007/s10388-022-00961-2, PMID: 36327058

[14] Sun SY Chen PP Meng LX Li L Mo ZX Sun CH . High preoperative plasma fibrinogen and serum albumin score is associated with poor survival in operable esophageal squamous cell carcinoma. Dis Esophagus. (2019) 32. doi: 10.1093/dote/doy057, PMID: 29905761

[15] Gao J Li M Wang Y Wang Z Chen X Li H . Prognostic Effect of the PNI and LSR in Patients with Esophageal Squamous Cell Carcinoma Patients Receiving Radiotherapy. J Gastrointest Cancer. (2024) 56:26. doi: 10.1007/s12029-024-01148-x, PMID: 39601941

[16] Zhang J Zhao P Xu R Han L Chen W Zhang Y . Comparison of the efficacy and safety of perioperative immunochemotherapeutic strategies for locally advanced esophageal cancer: a systematic review and network meta-analysis. Front Immunol. (2024) 15:1478377. doi: 10.3389/fimmu.2024.1478377, PMID: 39712027 PMC11659204

[17] Zhou N Hua Y Ge Y Wang Q Wang C He J . Perioperative tislelizumab with four cycles of neoadjuvant chemotherapy for resectable locally advanced esophageal squamous cell carcinoma: a phase 2 study. Front Immunol. (2024) 15:1482005. doi: 10.3389/fimmu.2024.1482005, PMID: 39687611 PMC11647006

[18] Ge Q Guo C Ma Y Li J Lian J Lu T . Comparative Efficacy and Safety of Neoadjuvant Immunochemotherapy Versus Chemotherapy in Locally Advanced Oesophageal Squamous Cell Carcinoma: A Dual-Centre Retrospective Study. Eur J Cardiothorac Surg. (2025) 67. doi: 10.1093/ejcts/ezaf268, PMID: 40754841

[19] Champiat S Ferrara R Massard C Besse B Marabelle A Soria JC . Hyperprogressive disease: recognizing a novel pattern to improve patient management. Nat Rev Clin Oncol. (2018) 15:748–62. doi: 10.1038/s41571-018-0111-2, PMID: 30361681

[20] Wei Z Zhang Y . Immune Cells in Hyperprogressive Disease under Immune Checkpoint-Based Immunotherapy. Cells. (2022) 11. doi: 10.3390/cells11111758, PMID: 35681453 PMC9179330

[21] Kwak JW Houghton AM . Targeting neutrophils for cancer therapy. Nat Rev Drug Discov. (2025) 24:666–84. doi: 10.1038/s41573-025-01210-8, PMID: 40374764 PMC13232631

[22] Bo Y Lu Q Li B Sha R Yu H Miao C . The role of platelets in central hubs of inflammation: A literature review. Med (Baltimore). (2024) 103:e38115. doi: 10.31219/osf.io/jghn7, PMID: 38728509 PMC11081549

[23] Zhang J Gu J Wang X Ji C Yu D Wang M . Engineering and Targeting Neutrophils for Cancer Therapy. Adv Mater. (2024) 36:e2310318. doi: 10.1002/adma.202310318, PMID: 38320755

[24] Ménétrier-Caux C Ray-Coquard I Blay JY Caux C . Lymphopenia in Cancer Patients and its Effects on Response to Immunotherapy: an opportunity for combination with Cytokines? J Immunother Cancer. (2019) 7:85. doi: 10.1186/s40425-019-0549-5, PMID: 30922400 PMC6437964

[25] Chen D Verma V Patel RR Barsoumian HB Cortez MA Welsh JW . Absolute Lymphocyte Count Predicts Abscopal Responses and Outcomes in Patients Receiving Combined Immunotherapy and Radiation Therapy: Analysis of 3 Phase 1/2 Trials. Int J Radiat Oncol Biol Phys. (2020) 108:196–203. doi: 10.1016/j.ijrobp.2020.01.032, PMID: 32036004

[26] Wang JL Ma R Kong W Zhao R Wang YY . Lymphopenia in Esophageal Cancer: What Have We Learned? Front Oncol. (2021) 11:625963. doi: 10.3389/fonc.2021.625963, PMID: 33791213 PMC8006429

[27] Iasonos A Schrag D Raj GV Panageas KS . How to build and interpret a nomogram for cancer prognosis. J Clin Oncol. (2008) 26:1364–70. doi: 10.1200/JCO.2007.12.9791, PMID: 18323559

