# Supplementary material for: Prediction of clinically significant complication following neoadjuvant chemoimmunotherapy in resectable esophageal cancer: a dynamic systemic inflammatory biomarker-based model
Source: Front Immunol. 2026 Mar 25;17:1719339. doi: 10.3389/fimmu.2026.1719339 (PMC13057385; doi:10.3389/fimmu.2026.1719339)
Supplement: Supplementary file 1 [file Table1.docx]

Supplementary Table 1. Comparison of treatment-related adverse events between in the two groups of patients.

| Adverse events, n (%) | NSC group  (n=212) | | | CSC group  (n=61) | |
| --- | --- | --- | --- | --- | --- |
|  | Grade 1-2 | Grade≥3 | Grade 1-2 | | Grade≥3 |
| Leukopenia | 101 (47.6) | 4 (1.9) | 29 (47.5) | | 2 (3.3) |
| Nausea or vomiting | 128 (60.4) | 5 (2.4) | 41 (67.2) | | 2 (3.3) |
| Anorexia | 136 (64.2) | 4 (1.9) | 42 (68.9) | | 1 (1.6) |
| Anemia | 72 (34.0) | 3 (1.4) | 22 (36.1) | | 1 (1.6) |
| Thrombocytopenia | 42 (19.8) | 1 (0.5) | 15 (24.6) | | 1 (1.6) |
| Fatigue | 48 (22.6) | 1 (0.5) | 15 (24.6) | | 2 (3.3) |
| Pneumonitis | 7 (3.3) | 0 (0) | 2 (3.3) | | 1 (1.6) |
| Aminotransferase increased | 25 (11.8) | 1 (0.5) | 11 (18.0) | | 1 (1.6) |
| Diarrhea | 13 (6.1) | 0 (0) | 4 (6.6) | | 0 (0) |
| Creatinine increased | 12 (5.7) | 0 (0) | 5 (8.2) | | 0 (0) |
| Hyperthyroidism | 17 (8.0) | 0 (0) | 6 (9.8) | | 0 (0) |
